# The Effect of Transcranial Direct Current Stimulation and Functional Electrical Stimulation on the Lower Limb Function of Stroke Patients

**DOI:** 10.3389/fnins.2021.685931

**Published:** 2021-09-21

**Authors:** Xiao-Hua Zhang, Tao Gu, Xuan-Wei Liu, Ping Han, Hui-Lan Lv, Yu-Long Wang, Peng Xiao

**Affiliations:** ^1^Department of Rehabilitation, Shenzhen Dapeng New District Nanao People’s Hospital, Shenzhen, China; ^2^Department of Rehabilitation, The First Affiliated Hospital, Shenzhen University, Shenzhen Second People’s Hospital, Shenzhen, China

**Keywords:** transcranial direct current stimulation, stroke, somatosensory evoked potential, leg motor function, post-convalescent

## Abstract

**Objective:** This study aimed to research the effect of transcranial direct current stimulation (tDCS) and functional electrical stimulation (FES) on the lower limb function of post-convalescent stroke patients.

**Methods:** A total of 122 patients in the stroke recovery stage who suffered from leg dysfunction were randomly divided into two groups: a tDCS group (*n* = 61) and a FES group (*n* = 61). All patients received same routine rehabilitation and equal treatment quality, the tDCS group was treated with tDCS, while the FES group received FES. The lower limb Fugl-Meyer assessment (FMA), modified Barthel index (MBI), functional ambulatory category (FAC), and somatosensory evoked potential (SEP) were used to assess the patients at three different stages: prior to treatment, 4 weeks after treatment, and 8 weeks after treatment.

**Results:** The assessment scores for FMA, MBI, and FAC for the lower extremities after treatment (*P* > 0.05) were compared with those before treatment. The FMA, MBI, and FAC scores of the tDCS group were significantly higher than those of the FES group in all three stages (*P* < 0.05). The FMA, MBI, and FAC assessment scores of both groups were significantly higher after 4 weeks of treatment than that before treatment, and the scores after 8 weeks of treatment were significantly higher than those after 4 weeks after treatment (*P* < 0.05). The P40, N45 latencies decreased and the P40, N45 amplitudes increased, but there was no significant difference before treatment and after treatment (*P* >0.05), and there was no significant difference of the tDCS and FES groups before treatment and after treatment.

**Conclusion:** In conclusion, FMA, MBI, and FAC indicate that both tDCS and FES can significantly promote the recovery of a patient’s leg motor function and tDCS is more effective than FES in the stroke recovery stage. The application value of SEP in stroke patients remains to be further studied.

## Introduction

Globally, around 15 million people suffer from stroke every year: approximately one every 2 s. Subsequently, approximately 5 million stroke patients die each year and another 5 million are left permanently disabled (Sabut et al., 2013). Many reasons can cause stroke (Wańkowicz et al., 2019a,b). China ranks highest in stroke morbidity, with approximately 2 million new cases each year, and stroke is gradually becoming a major public health problem (Sabut et al., 2013). As a result of the injury caused to the central nervous system, patients who have suffered a stroke experience decreased muscle strength and balance and abnormal muscle tension, which results in a motion disorder (Huaping et al.). Due to the incongruity of the supporting phase of the lower limbs of the affected side and the healthy side, a decrease in step speed and frequency, and uneven distribution of load on each side, the quality and adaptability of a patient’s walking are reduced (Qing et al., 2014).

As lower limb motor function is necessary for a person’s daily life (Liberson et al., 1961) and an important aspect of functional independence (Xuemei et al., 2000), Turnbull et al. (1996) considered the recovery of lower limb function an important goal in the rehabilitation of post-stroke patients. Therefore, more effective ways of promoting lower limb function during rehabilitation treatment need to be researched to improve a patients’ quality of life. Using current rehabilitation technologies and evaluation approaches as a foundation, more effective rehabilitation treatments and objective evaluation indexes can be identified.

Transcranial direct current stimulation (tDCS) is a non-invasive method of brain stimulation that evokes a weak direct electric current (typically 1∼2 mA) via electrodes placed onto the skull (Zhenfen et al., 2018). It stimulates cortical nerve cells, resulting in changes to the activity and excitability of cortical neurons, which can induce functional brain changes. Clinical studies have shown that tDCS modulates physiological activity in multiple functional areas of the brain, thereby improving limb dysfunction (Kang et al., 2018). It therefore shows great potential as a non-invasive and effective brain function regulation technology for the treatment of chronic pain, neurological diseases, mental diseases, and other related diseases.

Functional electrical stimulation (FES) is a therapeutic approach that utilizes a low-frequency pulse current to stimulate a group of muscles through a preset schedule that triggers muscle movement or mimics normal autonomic movement, thereby improving or restoring the function of the stimulated muscle or muscle group. Due to its particularly therapeutic effect, it has drawn more and more attention from medical workers. Consequently, it has been used as a treatment method to improve the limb activity of patients with limb dysfunction, especially among post-stroke patients or those with a spinal cord injury (Howlett et al., 2015).

tDCS and FES are methods of physical therapy. Despite many clinical studies confirming their effectiveness, however, the current literature has focused on upper limb function and improvements of other dysfunctions. Most clinical rehabilitation evaluation scales are also unable to sufficiently reflect the objective functional condition of patients (Lu et al., 2008). Further, a comparison of the effects of tDCS and FES on lower limb function in post-stroke patients has not yet been undertaken. Therefore, this study aimed to explore the effect of tDCS on the lower limb function of patients during the stroke recovery period and compare its rehabilitation efficacy with that of FES. Additionally, somatosensory-evoked potentials (SEPs) were used to evaluate efficacy using a routine assessment scale, with the aim of identifying an effective treatment for limb dysfunction in post-stroke patients and a more objective evaluation index.

## Materials and Methods

### Participants and Groups

In this prospective study, 122 post-stroke hemiplegic patients were recruited from the Shenzhen Dapeng New District Nan’ao People’s Hospital between October 2018 and October 2019. The study was evaluated and approved by the hospital’s Ethics Committee (2020013), and all patients signed informed consent forms. Using a random distribution number table, the patients were randomly divided into two groups: a tDCS group (*n* = 61) and a FES group (*n* = 61).

Inclusion criteria: (1) diagnosed with stroke according to medical history, clinical symptoms, imaging materials, and pathological results (Chinese Society of Neuroscience, and Chinese Society of Neurosurgery., 1996); (2) aged 18–70 years; (3) first stroke; (4) unilateral brain lesions; (5) had a course of 15 days to 6 months; (6) stable condition and able to complete training instructions and assessments; (7) patients or family members agreed to participate in the study and signed informed consent forms.

Exclusion criteria: (1) declined to join any one group; (2) severe physical dyskinesia or inability to complete training; (3) other severe somatic diseases and inability to complete subsequent training; (4) severe cognitive disorders, epilepsy, or mental disorders; (5) local skin injury or inflammation with hyperalgesia in the stimulation area; (6) implantable electronic devices; (7) at risk for intracranial hypertension and bleeding.

### Treatment

The patients in both groups were treated with conventional drugs that nourished brain cells and improved circulation, as well as with routine rehabilitation, such as exercise therapy, physical therapy, and traditional therapy. The tDCS treatment was administered to the patients in the tDCS group, while FES treatment was administered to the patients in the FES group. The prescribed treatment time for both groups was 20 min each day for 5 days a week over a total of 8 weeks.

#### tDCS Treatment

A TC-100 transcranial direct current stimulator produced by Wuhan Yimai Medical Technology Enterprise was used. tDCS was delivered by a battery-powered constant current stimulator using a pair of surface saline-soaked sponge electrodes placed on the scalp. The size of the stimulation electrode is 5^∗^7 cm^2^. The anode was placed on the stimulation site in the central anterior motor area of the brain, and the cathode was placed on the contralateral forehead (according to the 10–20 electroencephalograph system for electrode placement). During active tDCS, a constant current of 2.0 mA was applied for 20 min, with a ramping period of 30 s at both the beginning and end of stimulation (i.e., fade-in and fade-out phases, respectively) (Inguaggiato et al., 2019). The stimulation protocol was 20 min each day for 5 days a week over a total of 8 weeks.

#### FES Treatment

A six-channel FES rehabilitation treadmill training system (FES rehabilitation treadmill intelligent training system, Yasi Company) equipped with multiple output channels was used. This system could simultaneously stimulate multiple muscle groups, resulting in coordinated movements of multiple joints (such as the knee and ankle joints), which more closely reflects the requirements of functional activities. FES stimulates a string of muscles, Standard surface electrodes (3 × 3 cm) were placed on the erector spine, gluteus maximus, quadriceps femoris, hamstring, tibialis anterior, and gastrocnemius muscles of the affected side. The indexes of the equipment were set individually, with a frequency of 15∼50 Hz, width of 200 and 300 μs, current stimulation intensity of 4∼20 mA. By adjusting the intensity, the electrodes are connected to the FES stimulator by means of a cable, and the intensity adjustment option is used to evaluate the contractile ability of each affected side muscle group according to the condition of different patients. Before the first training, patients would be tested for muscle current strength tolerance to determine the maximum and minimum current that can be tolerated by the muscles of the lower extremity on the affected side, so as to ensure that patients are always subjected to effective and safe current stimulation during the training process and avoid electric injury (Sharif et al., 2017). The stimulation protocol was 20 min each day for 5 days a week over a total of 8 weeks.

### Evaluation Criteria

All patients were tested with SEPs, Fugl-Meyer assessment (FMA), Modified Barthel index (MBI), and Functional ambulation category (FAC) by the same professionally trained physician, who was blinded to the different groups. The results were assessed by another physician at three stages: prior to treatment, 4 weeks after treatment, and 8 weeks after treatment.

### SEPs

The lower limb SEP results were collected using electromyography (German SIGMA Medical Technology Company, Neurowerk EMG). The test environment required the patient to be conscious and quiet. The stimulation point was the tibial nerve, which is the main area that collects P40 latency, P40 amplitude, N45 latency, and N45 amplitude in the lower limbs. The latent period and amplitude of each wave form before and after treatment were analyzed by EMG-evoked potentiometer software. Each stimulus was set to be superposed an average of 150 times. The analysis time was 100 ms, and measurement was completed twice (Xiaoyan et al., 2016). An average value was then taken as the final test result.

### Fugl-Meyer Assessment (FMA), Lower Extremity Portion of the Scale

The FMA is currently widely accepted throughout China because it is practical and reliable. It consists of 17 items, including motions with cooperative movement, motions without cooperative movement, reflex motions, hyperreflexia, coordination ability and speed, flexor-coordinated movement, and extensor-cooperative movement. Each item is divided into three grades (0, 1, and 2 points), with a maximum score of 34 points. The upper score is used to indicate improvement in each type of movement (Lindsay et al., 1990; Salazar et al., 2020).

### Modified Barthel Index (MBI)

The MBI reflects the basic aspects of a patient’s living situation, including hospital, their family, and their community. Because of its convenience, ease of use, high feasibility, credibility, and sensitivity, it is widely used as an evaluation tool in international rehabilitation medical institutions. It consists of 10 items, including bathing, toileting, eating, other personal hygiene, dressing, walking up and down stairs, and transferring from a bed to a chair. The maximum score is 100 points; the higher the score, the better the patient’s function (Shah et al., 1989; Collin et al., 1998).

### Functional Ambulation Category (FAC)

The FAC is scored on a scale of 0–5. 0 = patient cannot walk or needs the assistance of at least two other people; l = patient needs the assistance of one other person continuously to help them walk to lose weight and maintain balance; 2 = patient walks with one other person continuously or needs intermittent assistance; 3 = patient does not need direct physical support from other people, but still needs one other person’s supervision or verbal instruction; 4 = patient can walk independently on flat ground, but still needs other people’s help or support down slopes, stairs, or similar; 5 = patient is fully independent and can walk independently to anywhere they want (Hesse et al., 1999; Prodinger et al., 2017).

### Statistical Analysis

The data were processed using SPSS 22.0 statistical software. In this study, the measurement data were expressed as mean ± standard deviation (SD), Dose data sets between the independent samples *t*-test, set a significant level of *p <* 0.05. The categorical data were expressed as n (%), and analyzed by chi-square analysis or Fisher’s exact test. Comparison of the same group of patients before and after treatment was carried out using a paired student’s *t*-test or Mann-Whitney *U*-test, while comparison of the two groups after treatment was conducted using one-way analysis of variance (ANOVA). The regression equation was established, and repeated-measurement ANOVA was carried out. *P* < 0.05 was considered statistically significant.

## Results

### Baseline Data of Patients

A total of 122 patients were selected for this study. The FES group (*n* = 61) included 43 men (70.5%) and 18 women (29.5%), with an average age of 58.18 ± 11.70 years. The tDCS group (*n* = 61) included 38 men (62.3%) and 23 women (37.7%), with an average age of 56.11 ± 12.01 years. There were no significant differences between the two groups in gender or age, which means the background data were coincident with the subjects (*P* > 0.05) (Table 1).

**TABLE 1:** Comparison of patients’ baseline data.

Group	N	Gender (N)	Types of stroke	The average course of the disease (d, *x* ± *s*)	The average age (year, *x* ± *s*)	Location of cerebral injury (case)
		Male/Fe male	Cerebral hemorrhage/Cerebral infarction			Left/Right
FES group	61	43/18	19/42	(43.45 ± 5.66)	58.18 ± 11.70	27/34
tDCS group	61	38/23	22/39	(42.45 ± 4.75)	56.11 ± 12.01	30/31
t/χ^2^		0.918	2.758	1.382	0.962	0.056
P		0.338	0.431	0.171	0.338	0.813

### Leg Motor Function of Patients

**FIGURE 1:**
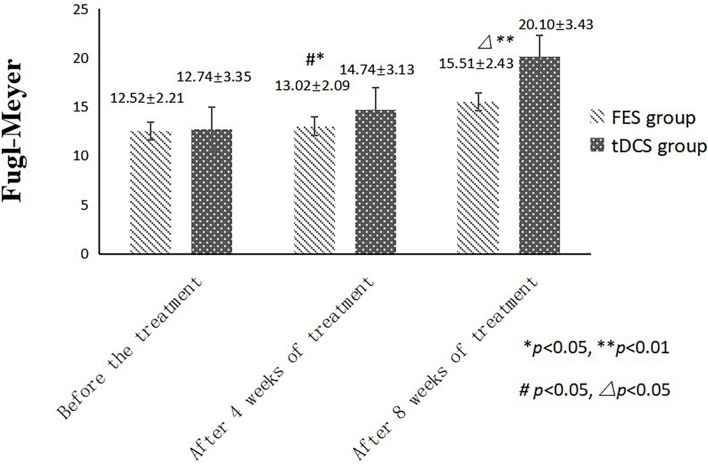
FMA scores comparing improvement before treatment, and after 4 and 8 weeks of FES and tDCS treatments in post-convalescent stroke patients. ^∗^Indicates that the mean value of the tDCS and FES groups is significantly different at the same time point, *p* < 0.05. ^∗∗^Indicates that the mean value of the tDCS and FES groups is significantly different at the same time point, *p* < 0.01. ^#^Indicates that the mean score after treatment is significantly different from that before treatment, *p* < 0.05. ^△^Indicates that the mean value difference at this time point is significant compared with that after 4 weeks of treatment, *p* < 0.05.

**FIGURE 2:**
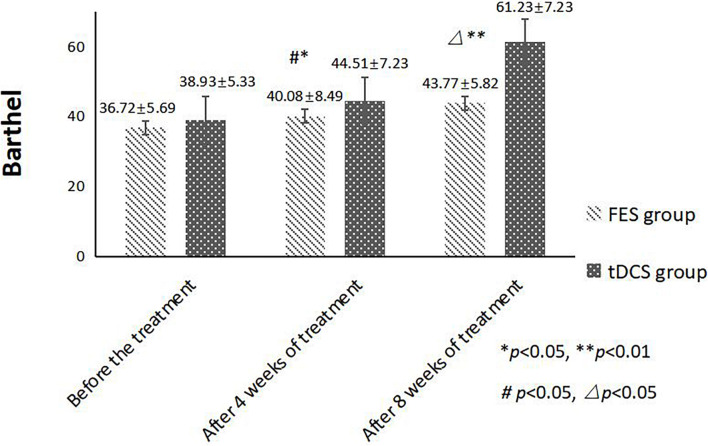
MBI scores comparing improvement before treatment, and after 4 and 8 weeks of FES and tDCS treatments in post-convalescent stroke patients. ^∗^Indicates that the mean value of the tDCS and FES groups is significantly different at the same time point, *p* < 0.05. ^∗∗^Indicates that the mean value of the tDCS and FES groups is significantly different at the same time point, *p* < 0.01. ^#^Indicates that the mean score after treatment is significantly different from that before treatment, *p* < 0.05. ^△^Indicates that the mean value difference at this time point is significant compared with that after 4 weeks of treatment, *p* < 0.05.

An independent sample *t*-test was used to compare the MBI and FMA indexes of the two groups, while repeated-measurement ANOVA was used to compare the results from the three treatment stages in the two groups. The difference in mean value of the FMA scores of the two groups was not statistically significant before treatment, but the score of the tDCS group was significantly higher than that of the FES group after 4 and 8 weeks of treatment. The mean difference values for each stage were 0.22, 1.72, and 4.59 between two groups, respectively. The difference in mean value of the Barthel indexes of the two groups was not statistically significant before treatment, but the score of the tDCS group was significantly higher than that of the FES group after 4 and 8 weeks of treatment, and the mean difference values of each stage were 2.21, 4.43, and 17.46, between two groups, respectively. All scores were significantly higher after 4 weeks of treatment than before treatment, and higher after 8 weeks of treatment than after 4 weeks of treatment (Table 2 and Figures 1, 2).

**TABLE 2:** The difference test before treatment, 4 weeks after treatment, and 8 weeks after treatment.

Group	Time	Barthel	Fugl-Meyer
FES group	Before the treatment	36.72 ± 5.69	12.52 ± 2.21
	After 4 weeks of treatment	40.08 ± 8.49^#^	13.02 ± 2.09^#^
	After 8 weeks of treatment	43.77 ± 5.82^#^^△^	15.51 ± 2.43^#^^△^
tDCS group	Before the treatment	38.93 ± 5.33	12.74 ± 3.35
	After 4 weeks of treatment	44.51 ± 7.23^*^^#^	14.74 ± 3.13^*^^#^
	After 8 weeks of treatment	61.23 ± 7.23^*^^#^^△^	20.10 ± 3.43^*^^#^^△^

^*^Indicates that the mean value difference between the FES group and the tDCS group is significant at the same time point, p < 0.05.

*^#^Indicates that the mean value difference at this time point is significant compared with that before treatment, p < 0.05.*

*^△^Indicates that the mean value difference at this time point is significant compared with that after 4 weeks of treatment, p < 0.05.*

**FIGURE 3:**
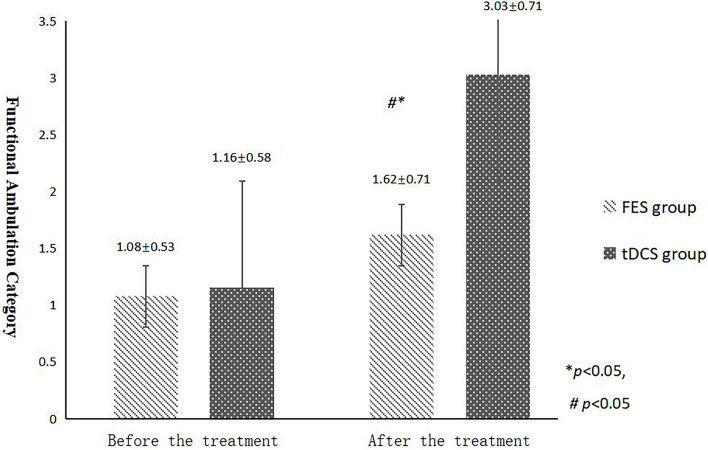
FAC scores comparing improvement before and after FES and tDCS treatments in post-convalescent stroke patients. ^∗^Indicates that the mean value of the tDCS and FES groups is significantly different at the same time point, *p* < 0.05. ^∗∗^Indicates that the mean value of the tDCS and FES groups is significantly different at the same time point, *p* < 0.01. ^#^Indicates that the mean score after treatment is significantly different from that before treatment, *p* < 0.05.

**FIGURE 4:**
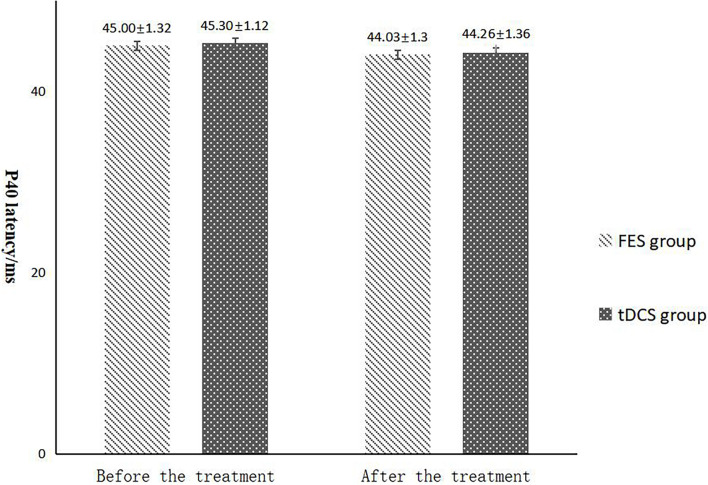
P40 latency comparing improvement before and after FES and tDCS treatments in post-convalescent stroke patients. ^∗^Indicates that the mean value of the tDCS and FES groups is significantly different at the same time point, *p* < 0.05. ^∗∗^Indicates that the mean value of the tDCS and FES groups is significantly different at the same time point, *p* < 0.01. ^#^Indicates that the mean score after treatment is significantly different from that before treatment, *p* < 0.05.

**FIGURE 5:**
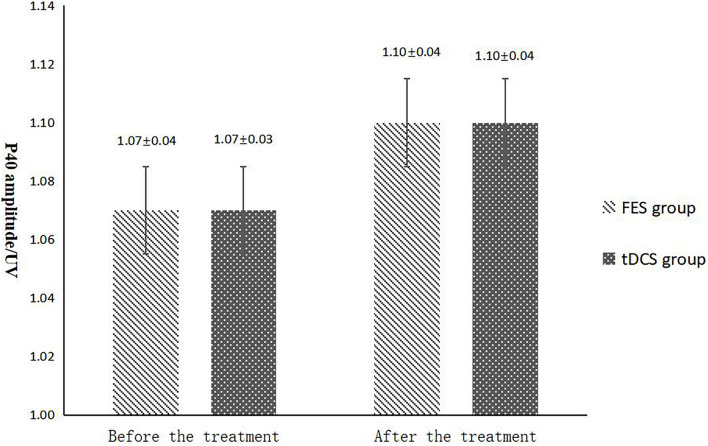
P40 amplitude comparing improvement before and after FES and tDCS treatments in post-convalescent stroke patients. ^∗^Indicates that the mean value of the tDCS and FES groups is significantly different at the same time point, *p* < 0.05. ^#^Indicates that the mean score after treatment is significantly different from that before treatment, *p* < 0.05.

**FIGURE 6:**
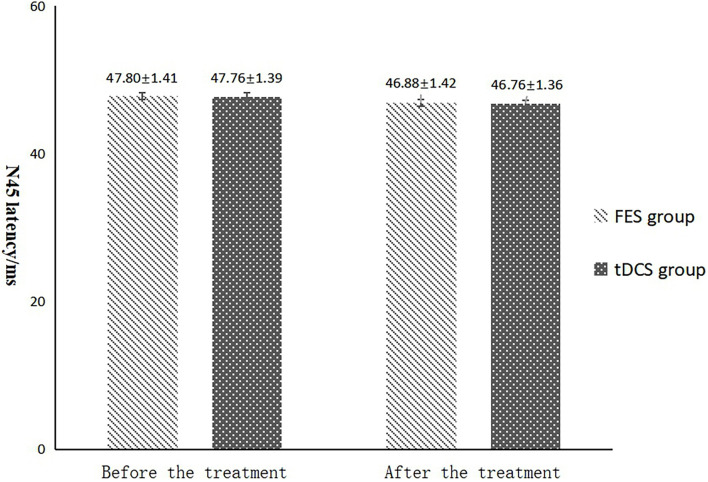
N45 latency comparing improvement before and after FES and tDCS treatments in post-convalescent stroke patients. ^∗^Indicates that the mean value of the tDCS and FES groups is significantly different at the same time point, *p* < 0.05. ^#^Indicates that the mean score after treatment is significantly different from that before treatment, *p* < 0.05.

**FIGURE 7:**
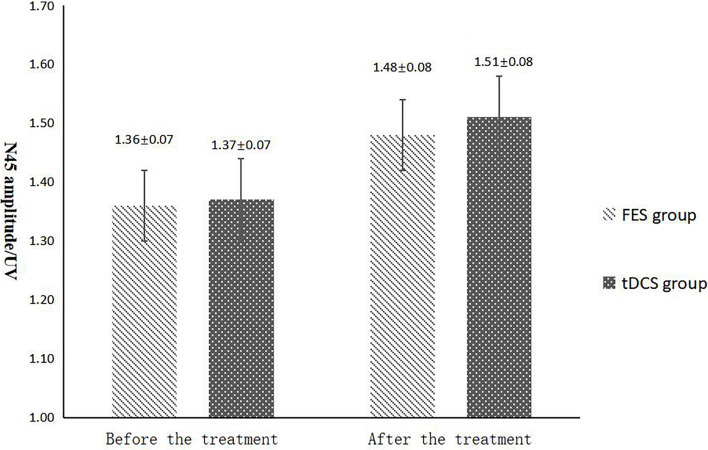
N45 amplitude comparing improvement before and after FES and tDCS treatments in post-convalescent stroke patients. ^∗^Indicates that the mean value of the tDCS and FES groups is significantly different at the same time point, *p* < 0.05. #Indicates that the mean score after treatment is significantly different from that before treatment, *p* < 0.05.

The mean FAC scores of the two groups were not significantly different before treatment, but the score of the tDCS group after treatment was significantly higher than that of the FES group. In addition, the mean scores of both groups after treatment were significantly higher than those before treatment. Compare within groups, the P40, N45 latencies decreased and the P40, N45 amplitudes increased, but there was no significant difference before treatment and after treatment (*P*> 0.05), and there was no significant difference of the tDCS and FES groups before treatment and after treatment (Table 3 and Figures 3–7).

**TABLE 3:** The difference in index scores before and after treatment.

Group	Time	FAC	P40 latency	P40 amplitude	N45 latency	N45 amplitude
FES group	Before treatment	1.08 ± 0.53	45.00 ± 1.32	1.07 ± 0.04	47.80 ± 1.41	1.36 ± 0.07
	After treatment	1.62 ± 0.71^#^	44.03 ± 1.30^#^	1.10 ± 0.04^#^	46.88 ± 1.42^#^	1.48 ± 0.08^#^
tDCS group	Before treatment	1.16 ± 0.58	45.30 ± 1.12	1.07 ± 0.03	47.76 ± 1.39	1.37 ± 0.07
	After treatment	3.03 ± 0.71^*^^#^	44.26 ± 1.36^#^	1.10 ± 0.04^#^	46.76 ± 1.36^#^	1.51 ± 0.08^#^

*Indicates that the mean value of the treatment and control groups is significantly different at the same time point, p < 0.05.

*^#^Indicates that the mean score after treatment is significantly different from that before treatment, p < 0.05.*

### Evaluation of Adverse Events

No serious adverse reactions, such as epilepsy, occurred in the two groups.

## Discussion

In recent years, the research of tDCS applied to motor dysfunction after stroke has focused on the upper limb, but less on the lower extremity motor dysfunction. Most of the studies were small sample size studies, and most of them were assessed by stroke assessment scales. These studies did not use neurophysiology, such as SEP, and/or neuroimaging techniques, such as functional magnetic resonance imaging (fMRI), to detect the changes of neurophysiological activities in the prognosis of TDCS (Tanaka et al., 2011; Sohn et al., 2013). The stroke assessment scale could be affected by subjective factors of the rater, and its predictive effect on functional recovery was not objective enough (Kang et al., 2016). SEP, as an objective neurophysiological examination, excludes the subjective influence of the examinee, which is more objective than stroke assessment scale, and is more practical for clinical evaluation of patients with aphasia and cognitive impairment (Hendridcs et al., 1997; Feys et al., 2005; Elsner et al., 2017).

At present, there is no standard paradigm for the stimulation course of tDCS in scientific research and clinical. The experimental methods, stimulation parameters, and intervention periods used in various studies are inconsistent (Kang et al., 2016).

At present, most of the treatment time was set as 4 weeks, while our treatment time was set as 8 weeks. The evaluation time points were before treatment, after 4 weeks of treatment, and after 8 weeks of treatment. Therefore, we can compare the treatment effect at different time points and evaluate the effect of treatment time. The comparison of the effects of tDCS and FES on lower limb function in post-stroke patients has not yet been undertaken. Therefore, this study aimed to explore the effect of tDCS on the lower limb function of patients during the stroke recovery period and compare its rehabilitation efficacy with that of FES. Additionally, SEP were used to evaluate efficacy using a routine assessment scale, with the aim of identifying an effective treatment for limb dysfunction in post-stroke patients and a more objective evaluation index.

These results indicate that both tDCS and FES can improve the walking function and motor function of the lower limbs of post-stroke patients, thus improving their ability to carry out the activities of daily life. However, the results of treatment with tDCS were better than the results of treatment with FES with regard to improvement of the lower limb function of patients engaged in stroke recovery. This may be related to the direct effect of tDCS therapy on the cerebral cortex, which changes the polarity of the membrane potential and the excitability of the cerebral cortex and promotes the functional connection of the pre-motion area, motor area, and sensory motor area of the affected cerebral hemisphere. These changes enable the functional reorganization of the related nerve synapses, thus improving a patient’s lower limb motor function and ability to carry out the activities of daily life.

On the basis of evaluating the FMA, MBI, and FAC indicators, SEP were used in the study group before treatment and after 8 weeks of treatment. SEP are the electrical responses recorded by the peripheral nerves of the somatosensory system on its sensory nerve conduction pathways after receiving appropriate stimulation. The recovery of SEP amplitude in stroke patients is significantly correlated with the recovery of motor function (Feys et al., 2000), which can help predict the prognosis. The reason may be that the proximity of the somatosensory pathway and the motor pathway, and the central nervous system is often affected at the same time, which can respond to the extent and location of damage to the motor nerve (Nelson et al., 1986; Ferri et al., 2001).

The results of statistical tests on the P40 incubation period, P40 amplitude, N45 latency, and N45 amplitude showed that, after 8 weeks of treatment, the P40 and N45 latencies decreased. The decrease of latency indicated that the nerve conduction velocity was enhanced and the function was improved, which was negatively correlated with FMA, MBI, and FAC. And the P40 and N45 amplitudes increased, which indicated that the function was better than that before treatment, and it was positively correlated with FMA, MBI, and FAC. However, compare within groups, the P40, N45 latencies decreased and the P40, N45 amplitudes increased, but there was no significant difference before treatment and after treatment (*P* > 0.05), and there was no significant difference of the tDCS and FES groups before treatment and after treatment. Although there was no significant difference in statistics, the *p*-value of the comparison between the groups was gradually close to 0.05, and the data of P40 and N45 were improved than that before treatment. It may be related to the insufficient sample size. This may be also due to the measured value of latencie and amplitude being related to the location of the lesion in the brain, the size of the lesion, and patient height, age, Temperature and other factors. This suggests that a patient’s height, age, lesion location, Temperature and other factors should be fully considered when using latencie and amplitude assessment in order to minimize deviation. Since SEP is affected by many interfering factors and the neurogenesis source of some SEP waveforms is still unclear, as an auxiliary diagnosis and treatment method for stroke, the clinical application of SEP should be combined with the clinical manifestations and other examination results of patients to make a comprehensive and objective judgment. The studies by CHAESH et al. KIMSH et al. CHOISM et al. showed consistent results (Sutter et al., 2012; Chae et al., 2013; Horn and Tjepkema-Cloostermans, 2017).

FES refers to the use of a low-frequency pulse current to induce muscle movement to simulate a patient’s normal autonomous movement, with the purpose of improving strength in muscles and muscle groups. Experimental results have shown that FES therapy can improve the motor function of the lower limbs during stroke recovery (Francisco and Barrio, 2016; Eraifej et al., 2017). In recent years, the neural rehabilitation treatment program has been continuously developed and improved, although the peripheral rehabilitation treatment program has been unable to meet the needs of stroke patients with limb dysfunction and cannot provide most stroke patients with a great extent of motor function recovery. The present study found that tDCS therapy was more effective than FES therapy at improving lower limb function in stroke patients. During the study period, none of the patients had serious adverse reactions, and only a small number experienced mild discomfort. Therefore, the use of tDCS for the treatment of lower extremity motor dysfunction after stroke is safe, efficacious, and results in fewer adverse reactions. As a central intervention treatment method, tDCS should be integrated into clinical rehabilitation protocols to meet the rehabilitation needs of the majority of stroke patients. Shaheiwola et al. (2018) concluded that the combined use of tDCS and FES can facilitate improvements in upper extremity motor abilities in severe chronic stroke patients and is more beneficial than the protocol with FES therapy alone. However, few studies reported the efficacy of combined use on the lower limbs function improvement. Our group will observe the efficacy of tDCS combined with FES on lower limbs function improvement in the future study.

As the SEP latency of each wave is relatively constant, neurogenesis sources can be derived from the wave latency of each SEP. Currently, SEP peak latency is used as the localization and qualitative evaluation index (Song et al., 2016). However, at present, studies on the correlation between the injury degree of specific brain injury sites and clinical evaluation scale and the latency and amplitude changes of SEP waveforms are still relatively lacking. The follow-up studies on SEP changes in stroke patients in the rehabilitation process lack of multi-center, large sample size and long-term observation, either. Therefore, the application value of SEP in stroke patients remains to be further studied (Sutter et al., 2012; Chae et al., 2013; Horn and Tjepkema-Cloostermans, 2017).

The present study has some limitations. Firstly, the acute or sequelae period of stroke recovery and the effect of comparison problems need more large-scale clinical exploration. Secondly, the study did not conduct long-term follow-ups with the participants and therefore did not observe the long-term effects of the treatments. Future studies should compare the effects of different stages of stroke and determine whether the effects of treatment are related to the location of the lesion.

## Conclusion

In conclusion, both tDCS and FES can significantly promote the recovery of a patient’s leg motor function, and tDCS is more effective than FES in the stroke recovery stage. The application value of SEP need to be further studied.

## Data Availability Statement

The original contributions presented in the study are included in the article/supplementary material, further inquiries can be directed to the corresponding author/s.

## Ethics Statement

The studies involving human participants were reviewed and approved by the Shenzhen Dapeng New District Nanao People’s Hospital Ethics Committee. The patients/participants provided their written informed consent to participate in this study. Written informed consent was obtained from the individual(s) for the publication of any potentially identifiable images or data included in this article.

## Author Contributions

X-HZ and TG conceived the idea and conceptualized the study. PH collected the data. H-LL and X-WL analyzed the data. Y-LW drafted the manuscript. PX reviewed the manuscript. All authors read and approved the final draft.

## Conflict of Interest

The authors declare that the research was conducted in the absence of any commercial or financial relationships that could be construed as a potential conflict of interest.

## Publisher’s Note

All claims expressed in this article are solely those of the authors and do not necessarily represent those of their affiliated organizations, or those of the publisher, the editors and the reviewers. Any product that may be evaluated in this article, or claim that may be made by its manufacturer, is not guaranteed or endorsed by the publisher.
